# Advances in Eye Tracking Technology: Theory, Algorithms, and Applications

**DOI:** 10.1155/2016/7831469

**Published:** 2016-09-27

**Authors:** Hong Fu, Ying Wei, Francesco Camastra, Pietro Arico, Hong Sheng

**Affiliations:** ^1^Chu Hai College of Higher Education, New Territories, Hong Kong; ^2^Shandong University, Jinan 250100, China; ^3^Department of Science and Technology, University of Naples Parthenope, Centro Direzionale, Isola C4, 80134 Naples, Italy; ^4^University of Rome “Sapienza”, Piazzale Aldo Moro 5, 00185 Rome, Italy; ^5^Missouri University of Science and Technology, Rolla, MO, USA

The goal of eye tracking is to detect and measure the point of gaze (where one is looking) or the motion of eye(s) relative to the head. The eye tracking data obtained by an eye tracker provide new opportunities and potentials in a broad range of applications including human computer interaction, computer simulation/virtual reality, neuroscience, medical, and cognitive-behavioral research. In recent years, eye tracking technology has been undergoing rapid development with improvements in the accuracy, stability, and sampling rates. A number of technologies and techniques are now available, including head-mounted, glass, table-mounted, and embedded systems, and with these advances new opportunities and applications are emerging. This special issue aims to bring together theoretical and practical perspectives in the area of eye tracking technology to present and discuss the latest technological developments and to inspire interaction and creation.

The issue received fifteen submissions; each qualified submission was reviewed by two international reviewers that we warmly thank for their time and efforts. Seven papers have been accepted for the publication.

“Learning to Model Task-Oriented Attention” by X. Zou et al. describes a model of saliency based on bottom-up image features and target position feature. Experimental results demonstrate the importance of the target information in the prediction of task-oriented visual attention.

“Characterization of Visual Scanning Patterns in Air Traffic Control” by S. N. McClung and Z. Kang defines new concepts to systematically filter complex visual scanpaths into simpler and more manageable forms and develops procedures to map visual scanpaths with linguistic inputs to reduce the human judgement bias during interrater agreement. The developed concepts and procedures were applied to investigating the visual scanpaths of expert ATCs using scenarios with different aircraft congestion levels. The findings show that the scanpaths filtered at the highest intensity led to more consistent mapping with the ATCs' linguistic inputs, the pattern classification occurrences differed between scenarios, and increasing aircraft congestion caused increased scan times and aircraft pairwise comparisons. These results provide a foundation for better characterizing complex scanpaths in a dynamic task and automating the analysis process.

“EyeTribe Tracker Data Accuracy Evaluation and Its Interconnection with Hypothesis Software for Cartographic Purposes” by S. Popelka et al. introduced a possible combination of Hypothesis software with EyeTribe tracker. A new software platform was presented which connects eye tracking device with an experiment builder. Experimental results showed that the mixed research design combines the advantages of quantitative and qualitative methods.

“Low Cost Eye Tracking: The Current Panorama” by O. Ferhat and F. Vilariño provided an overview of remote visible light gaze trackers and challenges in this area. The authors also analyzed the explored techniques from various perspectives such as calibration strategies, head pose invariance, and gaze estimation techniques.

“Learning-Based Visual Saliency Model for Detecting Diabetic Macular Edema in Retinal Image” by X. Zou et al. presents a learning-based visual saliency model method for detecting diagnostic diabetic macular edema regions of interest in retinal image. The method introduces the cognitive process of visual selection of relevant regions that arises during an ophthalmologist's image examination. The proposed method outperforms state-of-the-art saliency models and salient region detection approaches derived for natural images.

“Real-Time Control of a Video Game Using Eye Movements and Two Temporal EEG Sensors” by A. N. Belkacem et al. presents an algorithm able to classify six classes of eye movement by using only two temporal EEG electrodes, thus, in a noninvasive way. Moreover, this algorithm has been tested on real-time applications, in particular the control, by means of the eye movements, of a screen cursor and then of a character in a video game. Results showed that the proposed algorithm had an efficient response speed demonstrating its efficacy and robustness in real-time control.

“Designs and Algorithms to Map Eye Tracking Data with Dynamic Multielement Moving Objects” by Z. Kang et al. presents algorithms to address the eye tracking analysis issues when participants interrogate dynamic multielement objects and when eye trackers are incapable of providing exact eye fixation coordinates. The approach was tested in air traffic control (ATC) operations and the more accurate results could be obtained for eye tracking data analysis.

We hope that the readers of this journal will find in the issue interesting papers and that this can encourage and foster further research on eye tracking technology.

*Hong Fu* *Ying Wei* *Francesco Camastra* *Pietro Aricò* *Hong Sheng*

